# Risk of Recall Among Plastic Surgery Devices Cleared Through the FDA 510(k) Pathway

**DOI:** 10.1093/asjof/ojag126

**Published:** 2026-07-01

**Authors:** Ravi Dhawan, Alexandra Boric, Orr Shauly, Troy Marxen, Anjali Om, Kendall Brooks, Gabriela García Nores, Albert Losken

## Abstract

**Background:**

The FDA 510(k) pathway is the predominant route by which medical devices reach the US market. Previous studies have examined recall risk across broad FDA panels, but none have characterized plastic surgery devices or compared recall patterns across subcategories within the field.

**Objectives:**

The aim of this study was to determine the rate and severity of FDA recalls among 510(k)-cleared plastic surgery devices, compare recall risk across subcategories, and benchmark against all other 510(k) devices.

**Methods:**

All 510(k)-cleared devices (2008-2023) were obtained from the FDA premarket notification database. Two reviewers classified 234 surgery panel product codes, identifying 103 plastic surgery codes (3194 devices) in 8 subcategories. Each device was matched to the FDA recall database by its 510(k) number. All remaining 510(k) devices (*n* = 45,182) served as comparator. Recall rates were compared using χ^2^ tests and risk ratios (RR); time to first recall was modeled with Cox proportional hazards regression with Bonferroni correction.

**Results:**

Of 3194 plastic surgery devices, 197 (6.2%) were recalled, including 11 Class I recalls (0.3%). The recall rate for all other 510(k) devices was 11.4% (RR, 0.54; 95% CI, 0.47-0.62; *P* < .001). After Bonferroni correction, 4 subcategories had elevated recall hazard vs lasers and energy: implants and fixation (hazard ratio [HR], 3.64), negative pressure wound therapy (HR, 2.53), surgical mesh and acellular dermal matrices (HR, 2.46), and wound closure (HR, 2.01).

**Conclusions:**

Plastic surgery 510(k) devices had a lower recall rate than nonplastic surgery devices, although risk varied substantially across subcategories. These data provide a specialty-specific baseline for postmarket device surveillance.

**Level of Evidence: 5 (Therapeutic):**

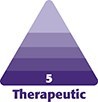

Plastic surgeons rely on an extensive array of medical devices in daily practice, from negative pressure wound therapy (NPWT) systems and acellular dermal matrices (ADMs) used in breast reconstruction to energy-based devices for skin resurfacing and laser-assisted lipolysis. The safety of these devices is of direct consequence to patient outcomes, yet the vast majority reach the market through the US FDA 510(k) clearance pathway, which requires a demonstration of substantial equivalence to an existing predicate device rather than independent clinical evidence of safety and effectiveness.^1-3^ Unlike Class III devices such as breast implants, which undergo rigorous premarket approval (PMA) with clinical trials, the Class II devices that constitute the overwhelming majority of the plastic surgeon's armamentarium are cleared on the basis of bench testing and descriptive data alone.^1,2,4^ Whether these devices are safe and how often they are recalled after reaching the market has not yet been thoroughly examined within the field of plastic and aesthetic surgery.

The FDA classifies medical devices into 3 risk categories: Class I (low risk, eg, tongue depressors), Class II (moderate risk, eg, surgical mesh), and Class III (high risk, eg, implantable pacemakers).^1,2^ The 510(k) pathway was conceived as a route for Class II products, permitting manufacturers to bring to market new products by showing substantial equivalence to a legally marketed predicate.^2,3^ Critics warn the pathway allows for “predicate daisy-chaining,” in which each successive generation of devices may bear little resemblance to the original.^5,6^ Zargar and Carr demonstrated that 97% of surgical meshes cleared between 2013 and 2015 trace back to just 6 pre-1976 devices, and 16% are linked to predicates that had themselves been recalled.^5^ A 2011 Institute of Medicine review judged substantial equivalence inadequate as a safety guarantee and called for its replacement.^6,7^

Multiple authors have used recall as a surrogate endpoint for device safety.^2,8-12^ In a landmark cohort study, Dubin et al analyzed 28,556 devices cleared or approved between 2008 and 2017 using a time-to-event framework and found, contrary to prevailing assumptions, that PMA devices carried 2.7 times the hazard of any recall and 7.3 times the hazard of Class I recall compared with 510(k) devices.^2^ This finding reflected the inherently higher-risk profile of Class III devices rather than a deficiency in the PMA pathway. However, because over 99% of devices enter the market through 510(k), the absolute burden of recalled 510(k) devices remains substantial.^2^ Further research showed that predicate device age and the number of ongoing predicate recalls have increased over time, suggesting growing risk for descendant devices.^13^ Others have demonstrated that devices citing recalled predicates are themselves at higher risk of subsequent recall.^14^

Importantly, Dubin et al reported that the “general and plastic surgery” panel had a significantly lower recall hazard (hazard ratio [HR], 0.78; 95% CI, 0.62-0.96; *P* = .008) compared with orthopedics.^2^ This finding is encouraging but of limited practical value to the plastic surgeon, because the FDA's “general and plastic surgery” panel encompasses a heterogeneous mix of devices. A single HR for the entire panel provides little insight into which types of plastic surgery devices carry greater or lesser risk, nor does it distinguish between wound dressings, energy devices, surgical mesh, NPWT systems, or implantable hardware, categories that differ fundamentally in their complexity, mechanism of action, and clinical risk profile.

This gap in the literature has relevance for the aesthetic surgery community. Aesthetic surgeons routinely use 510(k)-cleared devices for body contouring, wound management, and implant-based procedures. The FDA's 2019 determination that no ADM has ever been cleared for breast surgery, making their widespread use in implant-based breast reconstruction entirely off-label, and has underscored the importance of understanding the postmarket safety profile of these products.^15^ Informed device selection and patient counseling require specialty-specific recall data that do not currently exist.

To our knowledge, no study has examined recall variation among plastic surgery devices. The present study addresses this gap by analyzing 3194 plastic surgery devices cleared through the 510(k) pathway over 16 years (2008-2023), classifying them into 8 clinically meaningful subcategories, and benchmarking their recall rates against all other 510(k) devices. The specific aims were to determine the overall rate and severity of FDA recalls among 510(k)-cleared plastic surgery devices, compare recall risk across device subcategories, and establish the first specialty-specific baseline for postmarket device surveillance in plastic surgery.

## METHODS

### Study Design and Data Sources

This was a retrospective cohort study of all devices cleared through the FDA 510(k) pathway between January 1, 2008, and December 31, 2023. The study followed the Strengthening the Reporting of Observational Studies in Epidemiology reporting guideline. Institutional review board approval was not required as the study used only publicly available, deidentified federal data with no human participants.

The primary data source was the FDA's premarket notification database, which contains clearance records for all 510(k) devices, including decision date, applicant, product code, review panel, and clearance statement.^16^ Recall data were obtained from the openFDA device recall and enforcement application programming interfaces (APIs), which aggregate recall events including recall date, classification (Class I, II, or III), and product descriptions.^17^

### Identification of Plastic Surgery Devices

The FDA assigns each device a standardized product code linked to a review panel. We identified all 234 unique product codes assigned to the surgery (SU) review panel. Two reviewers (R.D. and O.S.) independently classified each product code as plastic surgery-relevant or not, based on the FDA's official device description for that code. Discrepancies were resolved by consensus discussion. This process yielded 103 product codes classified as plastic surgery-relevant, encompassing 3194 unique devices.

These 103 product codes were organized into 8 clinical subcategories based on primary function and clinical application: lasers and energy (*n* = 1578), wound dressings (*n* = 645), wound closure (*n* = 322), surgical mesh and ADM (*n* = 279), NPWT (*n* = 174), tissue processing (*n* = 85), tissue markers (*n* = 70), and implants and fixation (*n* = 41).

A reference group consisting of all remaining 510(k) devices cleared during the same period (*n* = 45,182) was assembled from all FDA review panels. Plastic surgery devices (*n* = 3194) were excluded from this group to prevent contamination.

### Recall Data Matching

Each device was individually cross-referenced against the FDA recall database using its unique 510(k) number (*K*-number) to determine recall status, date of first recall, and recall classification. The FDA codifies recalls according to the severity of potential harm: Class I represents a reasonable probability of serious adverse health consequences or death, Class II represents a temporary or reversible health consequence, and Class III represents unlikely adverse health consequences.^18^ Adapting from previous methodologies, when multiple recall events occurred for a single device on the same date, these were counted as a single recall to avoid overcounting.^2^

For the reference group, batch queries were performed using the openFDA API. These queries returned binary recall status (recalled vs not recalled) without individual recall dates, which precluded time-to-event analysis for the reference group.

### Statistical Analysis

Overall recall rates were compared between plastic surgery devices and the reference group using the Pearson χ^2^ test with Yates continuity correction for the 2 × 2 contingency table. A risk ratio (RR) with 95% CI was calculated using the log method.

Within the plastic surgery cohort, time to first recall was defined as the interval between the FDA clearance date and the date of first-recall event for recalled devices, or between the clearance date and December 31, 2025, for nonrecalled devices (censored). The study thus provided 2 to 18 years of follow-up for recall detection. Kaplan–Meier survival curves were generated for each subcategory, and differences across all 8 subcategories were assessed using the multivariate log-rank test.

Cox proportional hazards regression was used to estimate HRs for each subcategory relative to lasers and energy, which was selected as the reference category because it was the largest subcategory (*n* = 1578) and had the lowest recall rate. Separate pairwise Cox models were fitted for each subcategory vs the reference. *P* values were adjusted for 7 pairwise comparisons using the Bonferroni correction (*α* = 0.05/7 = .0071). A Bonferroni-adjusted *P* value of <.05 was considered statistically significant and denoted with a dagger symbol (†). All tests were 2-sided. Statistical analyses were performed using Python 3.10.

Because recall dates were not available for the reference group, the overall comparison of plastic surgery vs all other 510(k) devices used an RR rather than a Cox HR. This methodological distinction is noted throughout.

## RESULTS

### Overall Recall Rates

**Figure 1.**
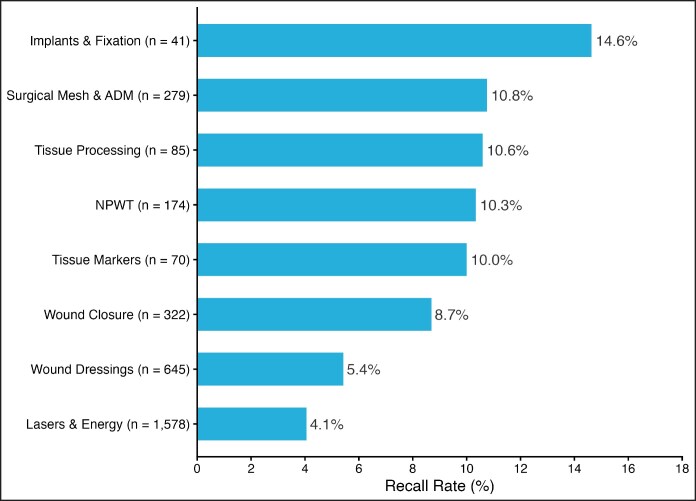
Recall rates by plastic surgery device subcategory. Horizontal bar chart showing the proportion of 510(k)-cleared plastic surgery devices (2008-2023) that received at least 1 FDA recall, stratified by subcategory. The 510(k) pathway is the most common FDA premarket notification process, through which manufacturers demonstrate that a new device is substantially equivalent to a legally marketed device. Recall rate was calculated as the number of devices with at least 1 recall divided by the total number of 510(k) clearances in each subcategory. Bars are sorted from lowest to highest recall rate, with sample size shown in parentheses. Across all 3194 plastic surgery devices, 197 (6.17%) were recalled. Implants and fixation had the highest subcategory recall rate (6/41; 14.6%) and lasers and energy had the lowest (64/1578; 4.1%). ADM, acellular dermal matrix; NPWT, negative pressure wound therapy.

During the 16-year study period, 48,376 devices received 510(k) clearance across all the FDA review panels. Of the 3194 plastic surgery devices, 197 (6.2%) were recalled, including 11 Class I recalls (0.3%). The recall rate for all other 510(k) devices was 11.4% (5158/45,182). Plastic surgery devices had a significantly lower recall rate than nonplastic surgery 510(k) devices (χ^2^ = 82.94; RR, 0.54; 95% CI, 0.47-0.62; *P* < .001; Figure 1, Table 1).

**Table 1.** Cohort Characteristics of 510(k) Plastic Surgery Devices and All Other 510(k) Devices, 2008-2023

Device group	*n*	Recalled	Rate (%)	Class I recalls	Class I recall (%)	Median FU, years
Lasers and Energy	1578	64	4.06	2	0.13	8.6
Wound Dressings	645	35	5.43	2	0.31	10.8
Wound Closure	322	28	8.70	0	0.00	9.6
Surgical mesh and ADM	279	30	10.75	1	0.36	10.8
NPWT	174	18	10.34	0	0.00	8.9
Tissue Processing	85	9	10.59	2	2.35	8.9
Tissue Markers	70	7	10.00	2	2.86	9.8
Implants and Fixation	41	6	14.63	2	4.88	10.2
Plastic surgery (total)	3194	197	6.17	11	0.34	9.4
All other 510(k)	45,182	5158	11.42	—	—	9.8
All 510(k) devices	48,376	5355	11.07	—	—	9.6

Recall status determined by cross-referencing each *K*-number against the openFDA device recall database. Class I data unavailable for aggregate nonplastic surgery devices. ADM, acellular dermal matrix; FU, follow-up; NPWT, negative pressure wound therapy.

### Recall Rates by Subcategory

Within-plastic surgery, recall rates varied from 4.1% (lasers and energy; 64/1578) to 14.6% (implants and fixation; 6/41). The intermediate subcategories were wound dressings (5.4%; 35/645), wound closure (8.7%; 28/322), tissue markers (10.0%; 7/70), NPWT (10.3%; 18/174), tissue processing (10.6%; 9/85), and surgical mesh and ADM (10.8%; 30/279; Table 2, Figure 1). Subcategories with the highest recall rates, implants and fixation, surgical mesh and ADM, and NPWT, generally involved devices that are implanted or that interface directly with open wounds for extended periods.

**Table 2.** Cox Proportional Hazards: Within-Plastic Surgery Subcategory Comparison

Subcategory	*n*	Events	HR (95% CI)	*P* value	*P* Bonf.
Implants and fixation	41	6	3.64 (1.57-8.40)	.002	.017†
NPWT	174	18	2.53 (1.50-4.28)	<.001	.003†
Tissue processing	85	9	2.47 (1.23-4.96)	.011	.078
Surgical mesh and ADM	279	30	2.46 (1.59-3.79)	<.001	<.001†
Tissue markers	70	7	2.28 (1.04-4.98)	.038	.269
Wound closure	322	28	2.01 (1.29-3.14)	.002	.014†
Wound dressings	645	35	1.20 (0.80-1.82)	.377	1.000
Lasers and energy	1578	64	1.00 [Reference]	—	—

Reference: Lasers and energy (largest subcategory, *n* = 1578). Events include number of devices with ≥1 recall (first-recall events), as in the Recalled column of Table 1. ADM, acellular dermal matrix; HR, hazard ratio; NPWT, negative pressure wound therapy. ^†^Significant after Bonferroni correction for 7 comparisons.

### Time-to-Recall Analysis

**Figure 2.**
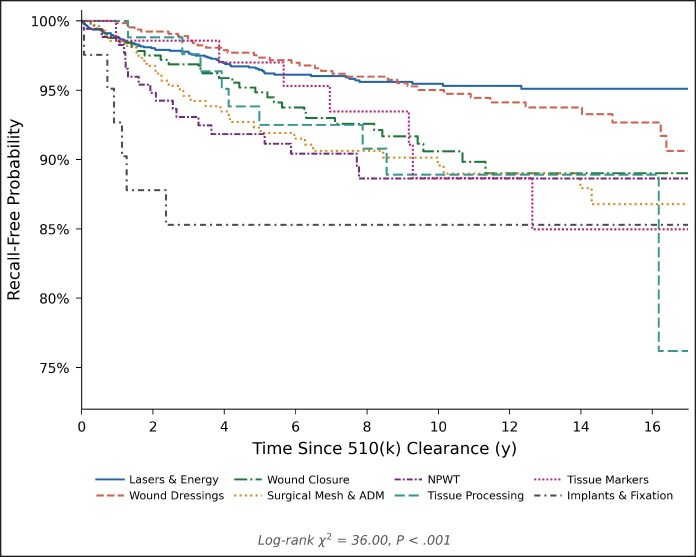
Kaplan–Meier survival curves for time to first recall by subcategory. Kaplan–Meier curves showing recall-free probability over time since 510(k) clearance for each of the 8 plastic surgery device subcategories. Time zero for each device is its individual 510(k) clearance date. The event of interest is the first FDA recall. Devices not recalled by the end of the study period (December 31, 2024) were censored at that date. A curve remaining near 100% indicates that most devices in that subcategory were not recalled over the follow-up period. Differences in recall-free survival across subcategories were assessed using the log-rank test, a nonparametric test that compares observed vs expected events across groups under the null hypothesis of equal hazard rates. The global log-rank χ^2^ = 36.00 (df = 7; *P* < .001). ADM, acellular dermal matrix; NPWT, negative pressure wound therapy.

Kaplan–Meier survival analysis demonstrated statistically significant differences in recall-free survival across the 8 subcategories (log-rank χ^2^ = 36.00, df = 7; *P* < .001; Figure 2). Implants and fixation, surgical mesh and ADM, and NPWT devices demonstrated the steepest declines in recall-free survival, with separation from the lasers and energy curve becoming apparent within the first 5 years after clearance. Wound dressings and lasers and energy demonstrated the most favorable survival trajectories.

### Cox Regression

**Figure 3.**
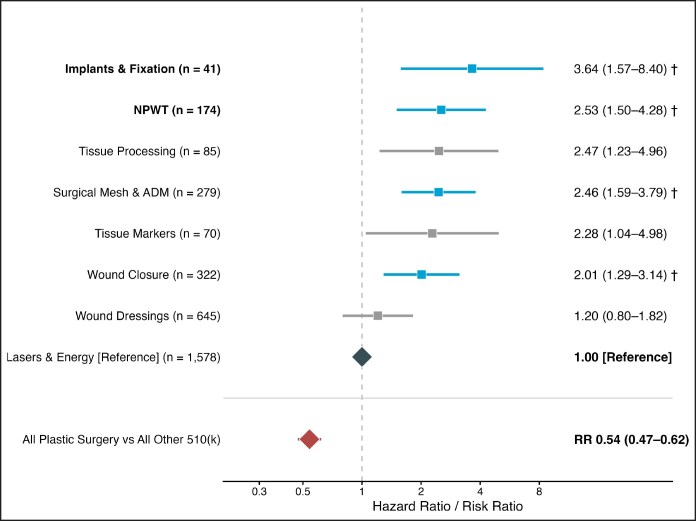
Forest plot of recall risk by device subcategory. Upper section: Hazard ratios (HRs) from a Cox proportional hazards regression model comparing the rate of first recall in each plastic surgery subcategory vs lasers and energy (reference; HR = 1.00). The Cox model accounts for differences in follow-up time across devices. Each square represents the point estimate; horizontal lines represent the 95% CI. Subcategories marked with † were statistically significant after Bonferroni correction for 7 simultaneous comparisons (adjusted significance threshold, *α* = 0.05/7 = .0071). Some subcategories have 95% CIs that do not cross 1.0 but lack the †symbol; these were nominally significant at unadjusted *P* < .05 but did not meet the Bonferroni-adjusted threshold. For example, tissue markers (HR, 2.28; 95% CI, 1.04-4.98) had an unadjusted *P* = .038 but a Bonferroni-adjusted *P* = .269. Lower section: Risk ratio (RR) comparing all plastic surgery 510(k) devices (*n* = 3194) with all other 510(k) devices (*n* = 45,182). Because recall dates were not available for the nonplastic surgery reference group (only binary recall status was obtainable from the FDA batch query system), a Cox model could not be applied for this comparison. Instead, an RR was calculated from a 2 × 2 contingency table and assessed with the Pearson χ^2^ test (χ^2^ = 82.94; *P* < .001). RR = 0.54 (95% CI, 0.47-0.62). ADM, acellular dermal matrix; NPWT, negative pressure wound therapy.

After Bonferroni correction for 7 comparisons, 4 subcategories demonstrated a significantly elevated hazard of recall relative to lasers and energy: Implants and fixation (HR, 3.64; 95% CI, 1.57-8.40; Bonferroni-adjusted *P* = .017), NPWT (HR, 2.53; 95% CI, 1.50-4.28; adjusted *P* = .003), surgical mesh and ADM (HR, 2.46; 95% CI, 1.59-3.79; adjusted *P* < .001), and wound closure (HR, 2.01; 95% CI, 1.29-3.14; adjusted *P* = .014; Table 3, Figure 3).

**Table 3.** Comparison of Recall Rates: Plastic Surgery vs All Other 510(k) Devices

	Plastic surgery (*n* = 3194)	All other 510(k) (*n* = 45,182)	All 510(k) (*n* = 48,376)	Risk ratio (95% CI)	*P* value
Any recall, *n* (%)	197 (6.17)	5158 (11.42)	5355 (11.07)	0.54 (0.47-0.62)	<.001
Class I recall, *n* (%)	11 (0.34)	—	—	—	—
Median follow-up, years	9.4	9.8	9.6	—	—

Risk ratio computed as plastic surgery rate/all other rate. 95% CI by log method. *P* value from χ^2^ test. Plastic surgery 510(k) devices had significantly lower recall rates than all other 510(k) devices.

Two additional subcategories, tissue processing (HR, 2.47; 95% CI, 1.23-4.96) and tissue markers (HR, 2.28; 95% CI, 1.04-4.98), had unadjusted *P* values <.05 and CIs excluding 1.0 but did not reach significance after Bonferroni correction (adjusted *P* = .078 and .269, respectively). Wound dressings (HR, 1.20; 95% CI, 0.80-1.82; adjusted *P* = 1.00) showed no significant difference from the reference.

### Class I Recalls

**Figure 4.**
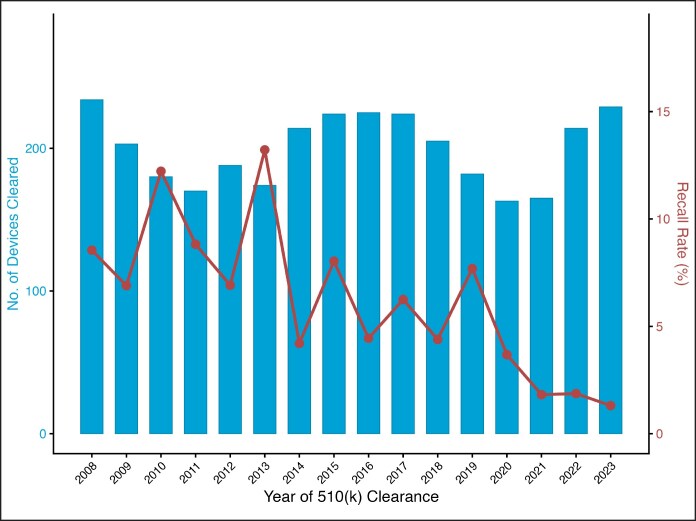
Annual 510(k) clearance volume and recall rate, 2008-2023. Dual-axis chart showing the annual number of plastic surgery 510(k) clearances (blue bars, left *y*-axis) and the corresponding recall rate (red line, right *y*-axis) for each clearance year. The recall rate for a given year was calculated as the number of devices cleared in that year that were subsequently recalled at any point during the study period divided by the total number cleared in that year. Annual clearance volume ranged from 163 (2020) to 234 (2008). The highest single-year recall rate was 13.2% (2013; 23/174 devices). Devices cleared more recently have shorter follow-up for recall detection; the declining recall rates observed in 2020-2023 may partly reflect this shorter observation period rather than a change in device safety.

**Figure 5.**
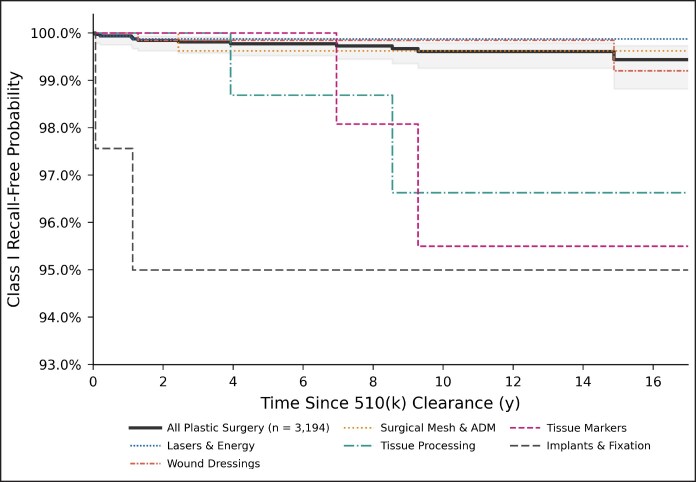
Kaplan–Meier survival curves for Class I recalls. Kaplan–Meier curves showing Class I recall-free probability over time since 510(k) clearance. Class I recalls are the most serious FDA recall classification, defined as situations with a reasonable probability of serious adverse health consequences or death. The black line with gray 95% CI ribbon represents all 3194 plastic surgery devices combined. Colored lines represent the 6 subcategories with at least 1 Class I event; wound closure (*n* = 322) and NPWT (*n* = 174) had zero Class I events and are not plotted individually. Across the entire cohort, 11 of 3194 devices (0.34%) received a Class I recall. The 95% CI ribbon widens at later time points because fewer devices remain under observation owing to shorter follow-up for more recently cleared devices. ADM, acellular dermal matrix; NPWT, negative pressure wound therapy.

Of the 197 recalled plastic surgery devices, 11 (5.6%) received Class I designations (0.34% of all 3194 devices). Class I recalls were concentrated in wound dressings (*n* = 2), tissue markers (*n* = 2), surgical mesh and ADM (*n* = 1), lasers and energy (*n* = 2), tissue processing (*n* = 2), and implants and fixation (*n* = 2). No Class I recalls were identified in the wound closure or NPWT subcategories (Figures 4, 5). Nonexhaustive, representative examples of recalled devices, including recall class, manufacturer, and reason for recall, are presented in Table 4.

**Table 4.** Representative Examples of Recalled 510(k)-Cleared Plastic Surgery Devices

Subcategory	Device	510(k) no.	Manufacturer	Class	Reason for recall	Year	Units in commerce
Implants and Fixation	Synthes Hemostatic Bone Putty	K103822, K113079	Synthes USA (DePuy Synthes)	I	Potential to ignite if contacted with electrosurgical cautery during surgery	2012	8853
Implants and Fixation	GEM FlowCoupler (4.0 mm)	K143589	Baxter Healthcare (Synovis)	II	IFU booklet may puncture outer Tyvek lid, compromising sterile barrier	2016	118
Surgical mesh and ADM	XenMatrix Surgical Graft (porcine dermal matrix)	K081272	Davol (C.R. Bard)	I	Could not guarantee product met FDA endotoxin limits; potential bacterial contamination	2011	1456
NPWT	Renasys Port Foam Dressing Kit	K082211	Smith & Nephew	II	Small holes in primary packaging pouch constituting breach of sterile barrier	2010	71,329
Wound closure	MONOCRYL Plus Antibacterial Suture	K201996	Ethicon (Johnson & Johnson)	II	Packaging machine defect resulted in hole in primary packaging; sterility compromised	2024	287,892
Lasers and energy	Leaseir MHR Xcell (810 nm diode laser)	K214049	Leaseir Technologies	II	Console label missing required DANGER symbol	2025	2
Tissue processing	Natrelle 133 Tissue Expander (BioCell textured)	K102806	Allergan	I	Higher incidence of BIA-ALCL associated with textured breast implant surface	2019	4,026,287

Data sourced from the FDA Recall Database (accessdata.fda.gov). Units in Commerce reflect the quantity reported by the manufacturer at the time of recall. Recalls are examples and do not reflect all recalled devices. Natrelle 133 was classified under tissue processing per the FDA product code LCJ (expander, skin, inflatable). Tissue expanders were grouped in this subcategory based on their primary function as temporary devices for tissue expansion rather than permanent implantation. ADM, acellular dermal matrix; BIA-ALCL, breast implant–associated anaplastic large cell lymphoma; IFU, instructions for use; NPWT, negative pressure wound therapy.

### Temporal Trends

Annual 510(k) clearance volumes for plastic surgery devices fluctuated modestly over the study period, with no clear secular trend. Recalled devices were distributed across all clearance years, although devices cleared in earlier years had longer follow-up and thus greater opportunity for recall events to be identified (Figure 4).

## DISCUSSION

This study represents the first analysis, to our knowledge, to identify plastic surgery devices from the FDA device panel and to characterize recall patterns across device subcategories. Among 3194 plastic surgery devices cleared through the 510(k) pathway over 16 years, the overall recall rate was 6.2%, which is substantially lower than the 11.4% recall rate for all other 510(k) cleared devices. However, considerable variation was observed across device subcategories, with implants and fixation devices demonstrating recall rates 3 times higher than those of lasers and energy devices.

Our finding that plastic surgery devices carry lower recall risk than the broader 510(k) universe is consistent with Dubin et al, who reported that the general and plastic surgery panel had a significantly lower hazard of recall (HR, 0.78) compared with orthopedics.^2^ Several factors likely contribute to this finding. The majority of plastic surgery 510(k) devices are relatively low in complexity, wound dressings, tissue markers, and surface-applied energy devices constitute over 70% of the cohort.^19^ Many are topical or external and are of temporary use, rather than permanently implanted, which reduces the severity profile and the likelihood of device-related adverse events triggering recalls.^2^ In contrast, specialties with higher recall rates, such as radiology (16.1%) and cardiovascular, tend to involve complex, often critical equipment that draws closer postmarket scrutiny.^2,20^

The subcategories with the highest recall rates, implants and fixation, surgical mesh and ADM, and NPWT, share a common feature in that they involve devices that are implanted or that maintain prolonged contact with compromised tissue. This pattern mirrors the broader literature. Dubin et al found that 29% of recalls are because of design deficiencies, which also account for over half of the most serious (Class I) events.^2^ Everhart et al also showed that risk of recall accumulates over time and device generations.^13^ The finding that implants and fixation devices had the highest recall rate (14.6%) despite being the smallest subcategory (*n* = 41) is notable. This subcategory includes plates, screws, and tissue anchors that are permanently implanted and subject to mechanical stress, paralleling the recall profiles seen in orthopedic surgery.^21^

### Surgical Mesh and the Regulatory Landscape

The surgical mesh and ADM subcategory merits particular attention given the current regulatory environment. These devices are cleared by 510(k) for general soft tissue repair indications, yet they are widely used in implant-based breast reconstruction, a use the FDA explicitly stated in March 2019 has never been cleared or approved.^15^ The FDA's position that ADMs require PMA for breast surgery has created what Boyd et al describe as a “catch-22”: obtaining PMA requires clinical trials in the breast indication, but manufacturers cannot promote the device for that use without approval.^15^ No manufacturer had secured ADM-specific PMA in breast reconstruction by early 2025, although investigational device exemption applications have been granted for prospective trials.^22^

Our finding that 10.8% of surgical mesh and ADM devices have been recalled, a rate more than double that of lasers and energy devices, adds urgency to these regulatory discussions. This rate is comparable to the 10.7% overall recall rate for orthopedic devices reported by Dubin et al.^2^ Other research has demonstrated that the predicate ancestry of surgical meshes is narrow and often interlinked with previous recalls, raising concerns about the propagation of design vulnerabilities.^5^ Plastic surgeons using ADMs in breast reconstruction should be aware that these products carry both off-label regulatory status and a recall rate that exceeds the average for plastic surgery devices.

### Breast Implants: A Notable Exclusion

It is important to note that breast implants, both silicone gel–filled and saline, are Class III devices approved through the PMA pathway and are therefore not included in this analysis.^23,24^ Breast implant safety has been extensively studied, with long-term postapproval follow-up of >99,000 participants having documented capsular contracture rates of 7.2% in primary augmentation, a 7-year reoperation rate of 11.7%, and the emergence of breast implant-associated anaplastic large cell lymphoma (BIA-ALCL) as a rare complication.^23^ The 2019 voluntary manufacturer recall of a macrotextured Biocell implants (Allergan, an AbbVie Company, Dublin, Ireland) for BIA-ALCL risk prompted substantial patient anxiety and changes in surgical decision making, with 49.5% to 59.5% of affected patients electing explantation or exchange.^24,25^ The PROFILE registry data show BIA-ALCL growing more common over the past decade, the age-adjusted rate reaching 26.9 per 100 million person-years by 2022, up from 14.5 a decade earlier.^26^ A future companion analysis examining recall patterns among PMA-approved plastic surgery devices, using the methodology described by Dubin et al for PMA supplements, would complement the present study.^27^

### Energy Devices and Negative Pressure Wound Therapy

Lasers and energy devices constituted the largest subcategory (*n* = 1578; 49.4% of all plastic surgery devices) and had the lowest recall rate (4.1%). This is consistent with the favorable safety profile reported in the dermatologic literature. A large multicenter prospective cohort found that fewer than 0.5% of cosmetic energy-based procedures resulted in an adverse event.^28^ Further research reported that the largest share of complications was attributable to provider use error (30%), with device malfunction the next most frequent contributor (20%).^29^

NPWT devices had a recall rate of 10.3% and an HR of 2.53 relative to lasers and energy. These devices interface directly with open or compromised wounds for extended periods, creating potential for adverse events related to seal integrity, pressure regulation, and biocompatibility. The SAWHI randomized clinical trial demonstrated comparable overall adverse event rates between NPWT and conventional wound treatment, although the NPWT group saw more maceration of the surrounding skin and local wound infection, supporting this reasoning.^19^

### Comparison With Other Specialties

The 6.2% overall recall rate for plastic surgery 510(k) devices compares favorably with most other specialties. Dubin et al reported recall rates of 16.1% for radiology, 13.5% for clinical chemistry, 12.2% for cardiovascular, and 10.7% for orthopedics.^2^ Specialty-specific analyses in otolaryngology have reported an 11.3% recall rate for 510(k) devices, whereas orthopedic studies report ∼12% recall rates within a decade of clearance, with a median time from clearance to Class I recall of 5.8 years for arthroplasty devices.^10,21^ Our finding that even the highest-risk plastic surgery subcategory (implants and fixation, 14.6%) falls within the range seen for other specialties suggests that plastic surgery devices as a group do not represent an outlier safety concern, but that specific subcategories warrant targeted surveillance.

### Implications for Practice

These data have several practical implications for plastic surgeons. First, the heterogeneity in recall rates across subcategories suggests that not all 510(k) devices carry equal risk, and clinicians should consider the recall history of specific device categories when making purchasing or treatment decisions. Second, the off-label status of ADMs in breast reconstruction, combined with their above-average recall rate, underscores the importance of informed consent discussions that acknowledge the regulatory context.^15^ Third, the low rate of Class I recalls overall (0.3%) is reassuring, although the concentration of these events in wound dressings and NPWT devices suggests that even seemingly low-risk device categories can occasionally pose safety concerns. Clinically, these subcategory-level data may inform device selection and patient counseling. This information does not replace clinical judgment but provides an evidence-based framework for preoperative informed consent discussions, particularly for off-label device uses where postmarket safety data are otherwise limited.

Plastic surgeons should also be aware of the MAUDE (Manufacturer and User Facility Device Experience) database as a reporting mechanism for adverse events. Underreporting is pervasive, the FDA acknowledges that MAUDE is a “passive surveillance system that has limitations, including the potential submission of incomplete, inaccurate, untimely, unverified, or biased data.”^30,31^ Across 9 years of data, MAUDE submissions prompted only 44 Medical Device Safety Communications, an initial indication of how passive reports are rarely converted to formal safety actions.^31^ Clinician engagement in adverse event reporting is essential for improving postmarket surveillance.^3,14^ Finally, nearly half of US health service areas lack a practicing plastic surgeon and only 19.5% of graduates practice in medically underserved areas; thus, device safety information must reach practitioners often in isolated practice settings rather than relying solely on institutional recall notification systems.^32^

### Future Directions

Several avenues for future investigation merit consideration. First, the present study was limited to devices cleared through the 510(k) pathway; a complementary analysis of Class III plastic surgery devices approved through the PMA pathway, including breast implants, dermal fillers, and tissue expanders, would provide a more complete picture of postmarket device safety across the full regulatory spectrum.^23,27,33^ Second, expanding this subcategory-level framework to other surgical specialties would enable direct benchmarking of recall risk between fields and help identify whether the patterns observed here are unique to plastic surgery or reflect broader trends in device safety.^5,27,34^ Third, as the FDA increasingly clears artificial intelligence (AI), and machine learning-enabled devices, including those with applications in surgical planning, image analysis, and intraoperative decision support, understanding the postmarket safety profile of these novel technologies will be essential, as their software-driven iterative modification may not be fully captured by traditional recall surveillance.^1,35-37^ Finally, although the present analysis focused exclusively on medical devices, a parallel examination of the FDA-regulated drugs and biologics used in aesthetic and reconstructive practice, such as botulinum toxin, or antibiotics, would complement these findings.

### Limitations

Several limitations should be considered. First, the classification of product codes as plastic surgery-relevant required subjective judgment. Although 2 reviewers independently classified each code with discrepancies resolved by consensus, some devices at the margins of plastic surgery practice (eg, general wound care products) could reasonably be classified differently. Second, recall is a surrogate endpoint that does not capture all device-related safety concerns. For example, the articular surface replacement metal-on-metal hip recall by DePuy Orthopaedics (subsidiary of Johnson & Johnson), despite being designated a Class II recall, had arguably a greater impact on public health than many Class I recalls of less widely used devices.^2,21^ Devices can also raise serious safety concerns without being formally recalled, as demonstrated by the Essure device (Bayer AG), which was voluntarily withdrawn from the market without a recall.^2^

Third, recall dates were not available for the reference group (all other 510(k) devices), which precluded a time-to-event comparison between plastic surgery and nonplastic surgery devices. The overall comparison, therefore, used an RR, which does not account for time on the market. Fourth, this study was limited to devices cleared through the 510(k) pathway and does not include PMA devices such as breast implants or injectable dermal fillers, which carry their own distinct risk profiles.^23,38^ Fifth, the FDA policy changes during the study period, including the staged implementation of Unique Device Identifiers from 2013 to 2020, may have an impact on recall rates and how they are counted, although Ghobadi et al found no significant increase in recalls associated with the passage of the Medical Device User Fee Amendments.^1,11,39^ Moreover, adverse events are more likely to be identified for devices used more frequently, a mediator in the causal relationship between device and recall that was not accounted for in this analysis or previous analyses on this subject.^2^ Finally, recalls are a surrogate marker for device safety: as this review shows devices can be recalled for packaging reasons, labeling, or regulatory scrutiny and do not directly reflect clinical failure.

## CONCLUSIONS

In this 16-year cohort study of 3194 510(k)-cleared plastic surgery devices benchmarked against 45,182 nonplastic surgery 510(k) devices, plastic surgery devices demonstrated a significantly lower overall recall rate (6.2% vs 11.4%; RR, 0.54; *P* < .001). However, recall risk varied substantially across device subcategories, with implants and fixation devices demonstrating more than 3 times the recall hazard of lasers and energy devices. These findings provide the first specialty-specific baseline for postmarket device surveillance in plastic surgery and may help inform device selection, patient counseling, and future regulatory oversight. Because the FDA continues to modernize the 510(k) pathway and evaluate the regulatory status of ADMs in breast surgery, specialty-specific recall data will be essential for evidence-based policy decisions.
